# Evaluating hepatitis C cascade of care surveillance system in Tuscany, Italy, through a population retrospective data-linkage study, 2015–2021

**DOI:** 10.1186/s12879-024-09241-z

**Published:** 2024-03-29

**Authors:** Luca Ceccarelli, Giaele Moretti, Sara Mazzilli, Davide Petri, Ilaria Corazza, Caterina Rizzo, Ersilia Lucenteforte, Milena Vainieri, Chiara Seghieri, Lara Tavoschi, On behalf of OPT-HepaC consortium

**Affiliations:** 1 Department of Translational Research and New Technologies in Medicine and Surgery, University of Pisa, Pisa, Italy; 2 Management and Healthcare Laboratory, Sant’Anna School of Advanced Studies, Pisa, Italy; 3 Scuola Normale Superiore, Pisa, Italy; 4 Department of Clinical and Experimental Medicine, University of Pisa, Pisa, Italy

**Keywords:** HCV, DAA treatment, Linkage to care, HCV screening, HCV diagnosis, Evidence-based policy making

## Abstract

This comprehensive retrospective data-linkage study aimed at evaluating the impact of Direct-Acting Antivirals (DAAs) on Hepatitis C Virus (HCV) testing, treatment trends, and access to care in Tuscany over six years following their introduction. Utilizing administrative healthcare records, our work reveals a substantial increase in HCV tests in 2017, attributed to the decision to provide universal access to treatment. However, despite efforts to eradicate chronic HCV through a government-led plan, the target of treating 6,221 patients annually was not met, and services contracted after 2018, exacerbated by the COVID-19 pandemic. Key findings indicate a higher prevalence of HCV screening among females in the 33–53 age group, influenced by pregnancy-related recommendations, while diagnostic tests and treatment uptake were more common among males. Problematic substance users constituted a significant proportion of those tested and treated, emphasizing their priority in HCV screening. Our paper underscores the need for decentralized HCV models and alternative testing strategies, such as point-of-care assays, especially in populations accessing harm reduction services, communities, and prisons. The study acknowledges limitations in relying solely on administrative records, advocating for improved data access and timely linkages to accurately monitor HCV care cascades and inform regional plans. Despite challenges, the paper demonstrates the value of administrative record linkages in understanding the access to care pathway for hard-to-reach populations. The findings emphasize the importance of the national HCV elimination strategy and the need for enhanced data collection to assess progress accurately, providing insights for future regional and national interventions.

**Supplementary Information:**

The online version contains supplementary material available at 10.1186/s12879-024-09241-z.

## Introduction

The hepatitis C virus (HCV) can cause acute and chronic hepatitis, potentially leading to the development of cirrhosis, liver cancer, and death [1–2]. Worldwide, an estimated 57.8 million people are chronically infected with HCV [3], of which 3.9 million are in the European Union/Economic European Area (EU/EEA). A large proportion of people chronically infected with HCV is undiagnosed, as chronic infection is often asymptomatic [4]. The burden of HCV is distributed unevenly in the population, including people in prison (PLP), people who use/have used drugs (PWUD), people living with HIV (PLHIV) and those with chronic diseases at increased risk of iatrogenic transmission (e.g., hemodialysis, diabetic patients, recipients of blood or other substances of human origin) being disproportionately affected [5].

To date, few studies have explored the epidemiology of HCV infections in the general population in Italy [6–11]. The limited evidence shows that an estimated 398,610 people in Italy live with active HCV infection (1.7% of the total population), with a higher prevalence in central regions (0.88%), followed by southern (0.72%) and insular areas (0.67%) and, lastly, northern regions (0.54%)[12, 13]. Currently, surveillance of viral hepatitis cases in Italy is focused on acute cases only [8] resulting in limited capacity of the system to estimate the burden of disease in the country. Large clinical cohort studies have also been set-up to support a variety of research activities, mostly on clinical grounds [12]. In very few instances, data linkage studies using administrative health records have been attempted to counter limited data availability. In Tuscany, a region of more than 3-million residents, a data-linkage study was conducted to assess the number of individuals with chronic HCV infection in need of treatment and to estimate the prevalence by the end of 2015. Based on records available and using a capture-recapture approach the resulting estimated HCV-RNA positivity prevalence was 1%, in line with other studies carried out in Italy [14].

The advent of direct-acting antivirals (DAA) led to the establishment of elimination targets by 2030, including a reduction in the undiagnosed fraction and increase in treatment coverage. To achieve this goal, the WHO has set several targets along the continuum of care for HCV, such as diagnosing 90% of those chronically infected and treating 90% of those in need by 2030 [15]. A Regional Action Plan was formulated by the European Office of the WHO, which adapts global targets to the regional epidemiological context and prioritizes actions to curb the disease [16]. The global and regional agenda for HCV elimination was integrated within the Italian National Plan for the Prevention of Viral Hepatitis (PNEV), endorsed by the Italian Ministry of Health in 2015 [17].

Free-of-charge DAA treatment at the point of care has been available in Italy since 2014 through specialists’ prescriptions and restricted to individuals with severe disease. The Italian Medicines Agency track new patients initiated on treatment through a monitoring registry for DAAs (https://www.aifa.gov.it/aggiornamento-epatite-c). Expanded access to DAA was achieved in Italy in 2017 [18]. Based on available estimate of disease, in Tuscany, a triennial action plan was launched in 2018 to increase the treatment coverage and contribute to the elimination of HCV in the region [19]. The plan foresaw more than 6000 HCV chronically infected individuals being treated every year up to 2020. In 2021, a national one-time screening program was launched [20], targeting PLP, PWUD and the 1969–1989 birth cohorts. However, the lingering COVID-19 pandemic has delayed its implementation across the country including Tuscany.

In order to assess the progresses against the HCV elimination goals in Tuscany region in Italy, we performed a retrospective data-linkage population study spanning over the 2015–2021 period. As a secondary aim, we described the strengths and weaknesses of a monitoring system consisting of administrative healthcare records.

## Materials and methods

### Setting and study population

The Italian health-care system is a regionally based National Health Service (NHS). The system is organized into three levels: national, regional and local. The national level is responsible for establishing strategies and policies. The regions are responsible for organizing and delivering health care services. Within each region, local health authorities (LHA) deliver public health services, community health services and primary care directly, and secondary and specialist care through either public hospitals or accredited private providers. The geographical area covered by each LHA is further divided into Health Care Districts (HCDs), which are responsible for the provision of public health and primary care services. The Italian health care system provides free care to every citizen who has a specific code for exemption from healthcare costs (exemption code). Exemption codes may be given to individuals after the diagnosis of a specific disease (e.g. HCV) or because of a particular socio-medical condition requiring tailored care and treatments (e.g. problematic drug use, imprisonment).

Tuscany is a region of central Italy that counts 3,673,347 inhabitants with three LHA (North-West, Center, South-East) and four university hospitals, to which 12 territories (provinces) refer. In Tuscany, about 90% of healthcare services are delivered by entities that are either public providers or private accredited providers. In the region there are 14 prisons, 40 harm reduction services (SerD) and 16 recognized non-governmental organizations (NGOs) that provide services to PWUD. The LHA (Ausl), 3 in Tuscany region, are territorial branches of the regional health service and guarantee the homogeneity of assistance in the different areas of the region. Divided into district-areas, they provide for the management and planning of the activities defined in the uniform and essential levels of care, including socio-medical services with high health integration, health services of social relevance and social assistance activities delegated to local authorities [21].

The Italian health care system waives out-of-pocket costs for citizens with certain health care needs through the attribution of exemption codes, which are unique alphanumeric codes assigned through an administrative process and recorded in the individual regional electronic record. Exemption codes may be given to individuals after the diagnosis of a specific disease (e.g. HCV) or for selected socio-medical conditions requiring tailored care and treatments (e.g. problematic drug use, detention). For the purpose of this study, we used exemption codes for the following conditions: chronic HCV infection (code 016); substance use disorders (code 014); incarceration (code F01).

### Administrative health records analysis

The Administrative health records are databases that track the delivery of healthcare services paid by the regional health system. Therefore, the system can track the single healthcare service, identified in the system by a unique code, provided to each individual person, identified in the system by a unique personal identifier (alphanumeric code). However, outcomes of the services provided (e.g., exams’ results, diagnostic procedures) are not available in the system in line with current national and regional privacy regulations.

Among the residents registered for healthcare services in the region, individuals who had been linked to at least one step of the hepatitis C care pathway between 2015 and 2021 were included in the study. These were individuals who had received a screening or diagnostic HCV test and/or a DAA prescription recorded in the health information system. Outpatient services data was used to identify patients that had, at least once in the study period, entered the HCV pathway via screening or through diagnostic test. Pharmaceutical services data contain information on drug prescriptions which was used to identify patients treated with DAA. Each patient in the dataset was identified, anonymously, through a unique code conferred by the regional agency for healthcare to ensure pseudo-anonymization.

A preliminary version of this algorithm was used in 2016 by some members of the research group. The algorithm was subsequently revised based on a recent study carried out in Tuscany [22]. In addition, through the use of condition-specific health care exemption codes, people who use drugs (PWUD) and people living in prison (PLP) were identified as priority groups for HCV testing and treatment. Subsequently, duplicates were eliminated by counting the same identification code that appeared in more than one service.

**Fig. 1:**
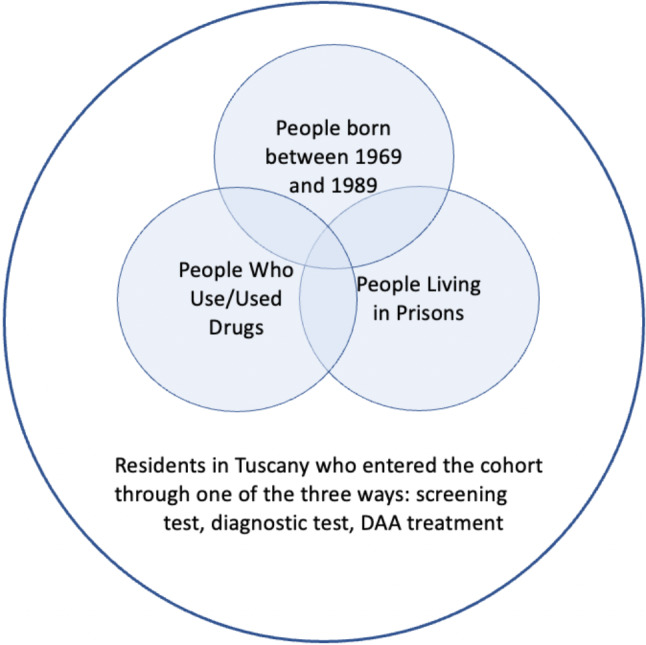
Definition of the study cohort per year and the three priority groups

Using the patient code, data linkage was performed, and the additional following information were collected for each individual included in the study population:

- sociodemographic characteristics: age, gender, specific exemption codes for problematic drug use and detention;
- screening by detection of HCV antibodies per year;
- diagnostic test by polymerase chain reaction (PCR) for HCV-RNA detection per year;
- treatment with DAA per year.

Of note, administrative health records capture the provision of a diagnostic or therapeutic service, but do not record diagnostic test results or clinical outcomes.

The database used for the description of HCV testing and treatment trends from 2015 to 2021 was built after data extraction from Tuscan Regional Health System health records using SAS 9.4 software. Supplementary Table 1 summarizes the type and characteristics of the variables.

Included patients were then divided into three age groups according to the target groups defined by the Italian Ministry of Health for the one-time screening intervention [23]: <33 years old, 33–53 years old, > 53 years old, in order to highlight the birth cohort 1969–1989. Additional groups for the analysis were PWUD and PLP (Fig. 1).

### Descriptive analysis

Our study analyzed trends in HCV screening coverage among residents in Tuscany, coverage of diagnostic test with PCR for HCV-RNA, and coverage of DAA treatment. A descriptive analysis of the main sample characteristics was conducted, stratifying data by sex and age. Data analysis was conducted for the general population group, defined as all individuals who had been linked to at least one step of the hepatitis C care irrespectively of particular risk categories or age groups. Data analysis was then repeated for the identified priority groups: birth cohort 1969–1989, PWUD, and PLP.

A three-step HCV care cascade (i.e., screening test for anti-HCV antibody, diagnostic test for HCV-RNA detection, and DAA treatment initiation) was used for this analysis. The annual percentage variation rate was calculated for each type of service: screening test, HCV-RNA test, and treatment. The percentage variation rate was calculated by taking the previous year as a reference for each year, starting in 2015 and ending in 2021.

Descriptive analysis was conducted using Stata/SE 15.1 by StataCorp LLC and Microsoft Excel 16.66.1.

## Results

### Overall results and trends

**Fig. 2:**
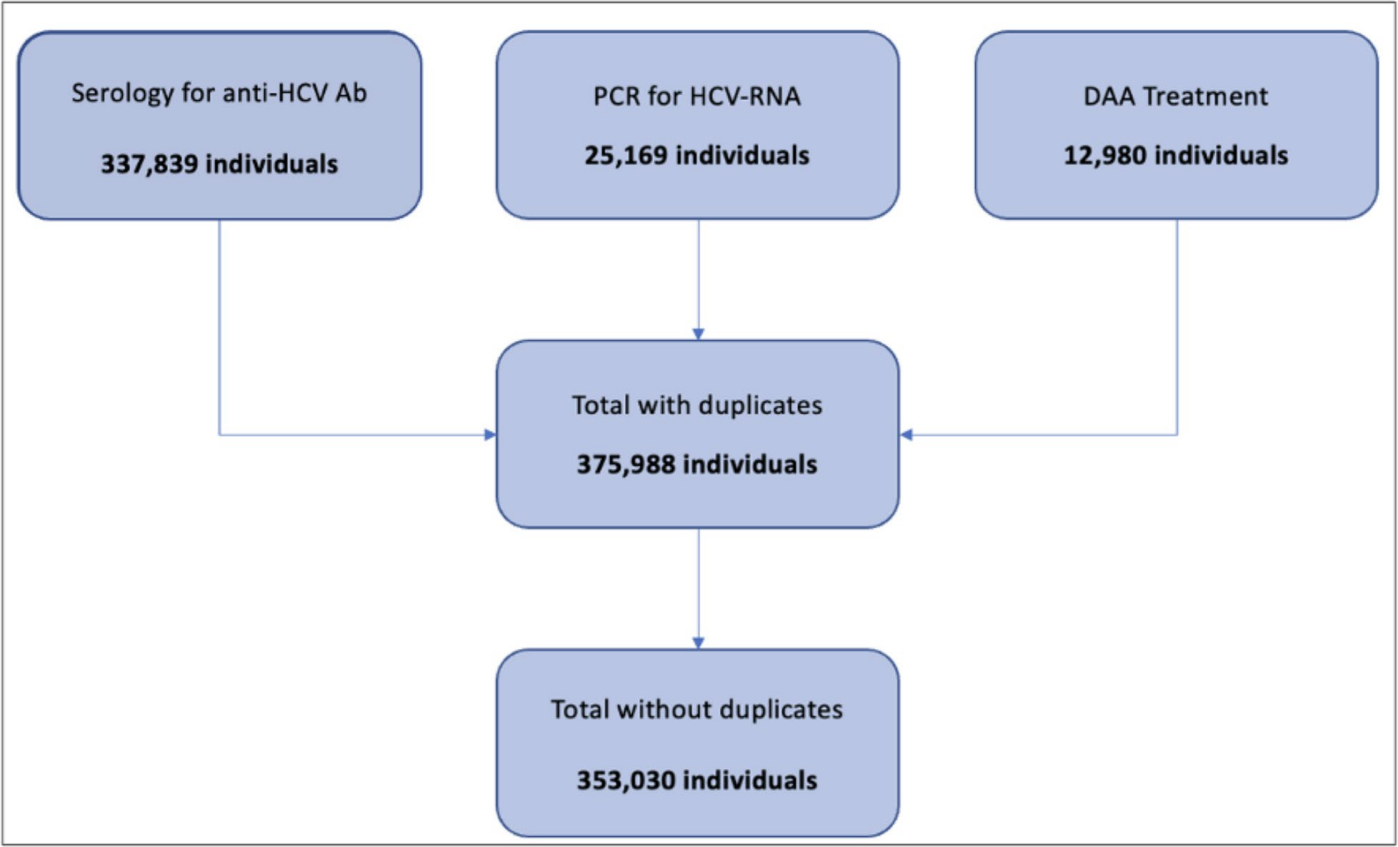
Data linkage

A total of 353,030 individuals (1.19% of the region inhabitants) were included in the study and had been linked to any step of the regional HCV pathway between 2015 and 2021. Of these, 216,180 were females (61.24%) (Fig. 2). The majority of the sample was in the 33–53 age group, which consisted of 156,115 individuals (44.22%), followed by the > 53 age group with 139,788 individuals (39.60%). The median age was 54 years for males (IQR 40–68) and 43 years for females (IQR 35–60), whereas the overall median age was 47 years (IQR 36–64). Within each age group, females predominated with 66.88% of the total in the < 33 age group, 68.97% in the 33–53 group and 51.62% in the > 53 group.

#### Screening and diagnosis – general population

**Fig. 3:**
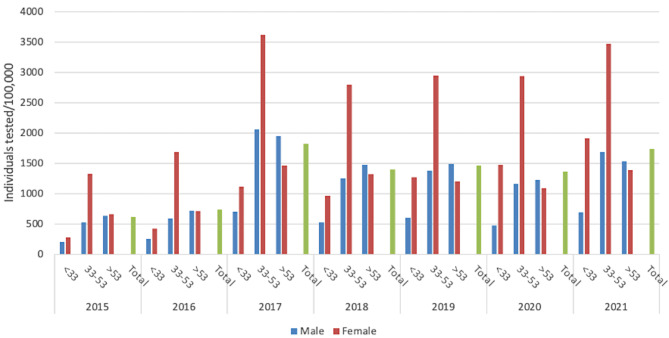
Individuals tested for HCV Ab by sex and age groups per 100,000 inhabitants, Tuscany, 2015–2021

From 2015 to 2021, in Tuscany a total of 337,839 people (1.14% of the entire regional population) were screened at least once for HCV through antibody testing (average 48,263/year) with a peak of 67,413 individuals in 2017. The testing rate in the general population was on average 1,300.29 tests per 100,000 population per year, with a minimum of 615.85/100,000 in 2015 and a maximum of 1,803.29/100,000 in 2017 (Fig. 3).

The total number of HCV screening tests performed by the regional health services was 434,801, resulting in 1.29 tests per person on average (1.31 for males; 1.34 for the age group 33–53). Of the general population screened (331,021 individuals), the majority (78.4%) were screened once, with 15.1%, and 6.5% screened twice and three times, respectively. Overall, 9.19% (152,811 individuals) of the general population residing in Tuscany as of 1 January 2022 had been tested at least once for HCV within the previous seven years.

Globally, females were tested more frequently than males (209,545 tests, 62.56%) as well as people within the 33–53 age group (152,628 tests, 45.18%).

A total of 25,169 (average 3,596/year, range 684-5,153) individuals were tested for HCV-RNA at least once between 2015 and 2021, corresponding to the 7.45% of those screened with anti-HCV Ab. Overall, in the study period, males (13,845 tests, 55.01%) and older individuals (> 53 years of age) were tested for HCV-RNA more frequently (17,186 tests, 68.28%).

#### Treatment – general population

**Fig. 4:**
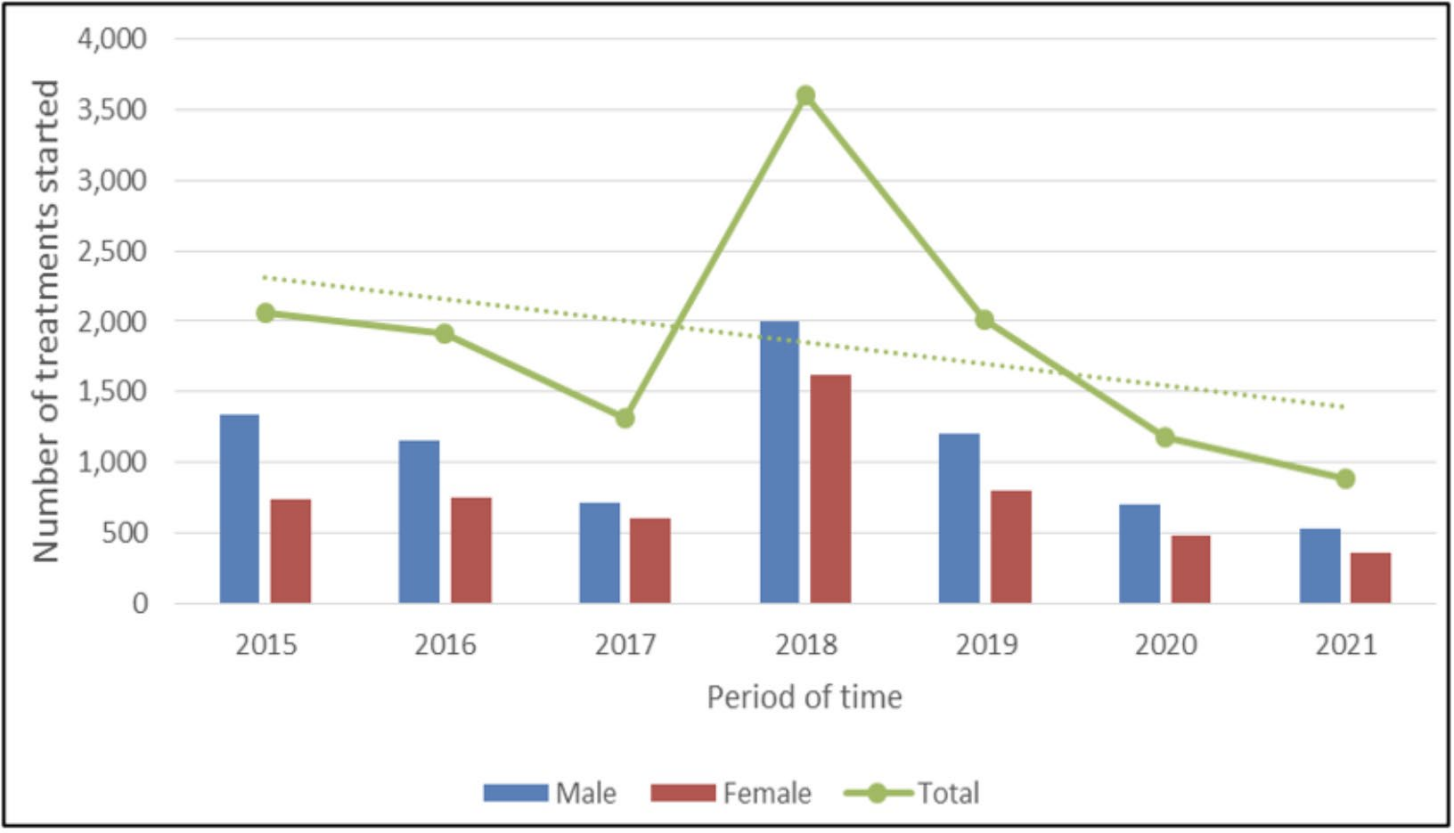
Number of DAA treatments initiated by sex, year of first prescription, and expected trend (dotted line) over time, Tuscany, 2015–2021; dotted line represents DAA treatments trend over the study period

A total of 12,980 patients (51.57% of all HCV-RNA tests performed) were prescribed DAA treatment, with an average of 1,854 individuals/year and a peak of 3,610 (27.81%) individuals in 2018. The majority of patients were aged > 53 years (9,737 treated, 75.02%) and were male (7,638 treated, 58.85%) (Fig. 4). Among all patients who started treatment, 9,428 (72.63%) had undergone an HCV-RNA PCR test in the previous calendar year.

### Detailed analysis per sub-population of interest

Among all patients who accessed the regional health care system for a service related to the HCV care cascade, the study focused on the population groups identified as priorities for national one-time screening: people born between 1969 and 1989, PWUD and PLP.

#### 1969–1989 birth cohort

Within the study cohort of people linked with HCV care, a total of 156,581 individuals (44.35%) were born between 1969 and 1989, thus making this population group more widely represented than the general population distribution: 14.49% of residents in Tuscany falling within the same birth cohorts. Of these, a total of 106,581 were females (68.27%). The median age was 40 for females, and 44 for males. A total of 152,811 individuals (45.23% of total screening tests performed) had an anti-HCV Ab screening test between 2015 and 2021, of whom 105,393 were females (68.97%), with a peak of 32,496 individuals tested in 2017. Out of those screened, 6,533 individuals had a HCV-RNA test, of whom 3,822 were males (58.50%).

Among individuals tested, a total of 2,975 individuals (1.95%) started treatment with DAAs, corresponding to 22.92% of all individuals treated during the study period. The peak was reached in 2018 with 967 treatments initiated, with an average of 425 per year.

#### People who use drugs (PWUD)

From 2015 to 2021, a total of 5,372 (1.52% of the study cohort) people who use drugs (PWUD) entered the HCV care pathway in Tuscany. Overall, the PWUD cohort consisted of 4,010 males (75.65%), with a median age of 45 years, and 1,362 females (23.35%), with a median age of 43 years.

As for screening, a total of 4,376 PWUD were tested for HCV antibodies (81.46%), with an average of 625 people tested each year (range 142-1,104). The age group in which the largest number of screening tests was performed was 33–53 years, and male individuals constituted the majority of PWUD tested. Additionally, 38.03% of PWUD (2,541 individuals) undertook the HCV screening test once in the study period, 20.04% twice, and 41.92% three or more times. Among PWUDs, the highest proportion of tests performed per person was recorded, with an average of 2.66.

Regarding HCV diagnosis, 1,779 PWUD were tested for HCV-RNA (40.65% of total PWUD), with an average of 254 people tested per year (range 40–556) and a peak in 2021. The average number of individuals tested for HCV-RNA was 192 (range 29–398) among males and 62 (range 11–158) among females in the study period.

A total of 1,249 PWUD started DAA treatment (70.21% of total PWUD tested for HCV-RNA), with an average of 178/year and a peak of 353 in 2018. Most treated patients belonged to the 33–53 age group (712 treated, 57.01%) and were male (992 treated, 79.42%). Among all PWUD who started the treatment, 889 (71.18%) had undergone an HCV-RNA PCR test in the previous year.

#### People living in prison (PLP)

From 2015 to 2021, a total of 4,954 (1.40% of the study cohort) people living in prison (PLP) entered the HCV care pathway through one of the related services. Overall, the PLP cohort consisted of 4,584 males (92.53%), median age 45 years, and 370 females (7.47%), median age 48 years.

As for screening, 4,277 PLP were tested for HCV antibodies (86.33% of total PLP), with an average of 611 people tested each year (range 148–852). The age group in which the largest number of screening tests were performed was 33–53 years, and male individuals constituted the majority of PLP tested. Additionally, 71.19% of PLP undertook the HCV screening test once in the study period, 17.33% twice and 11.05% three or more times.

In addition, 379 PLP were tested for HCV RNA (8.86% of total PLP tested for HCV RNA), with an average of 254 people tested per year (range 40–556) and a peak in 2021. A total of 111 PLP started treatment with DAA during the study period, corresponding to 29.29% of those tested for HCV RNA. Of these, 70 were in the 33–53 age group (63.06%) and 107 were males (96.40%).

### Cascade of HCV care and treatment

**Fig. 5:**
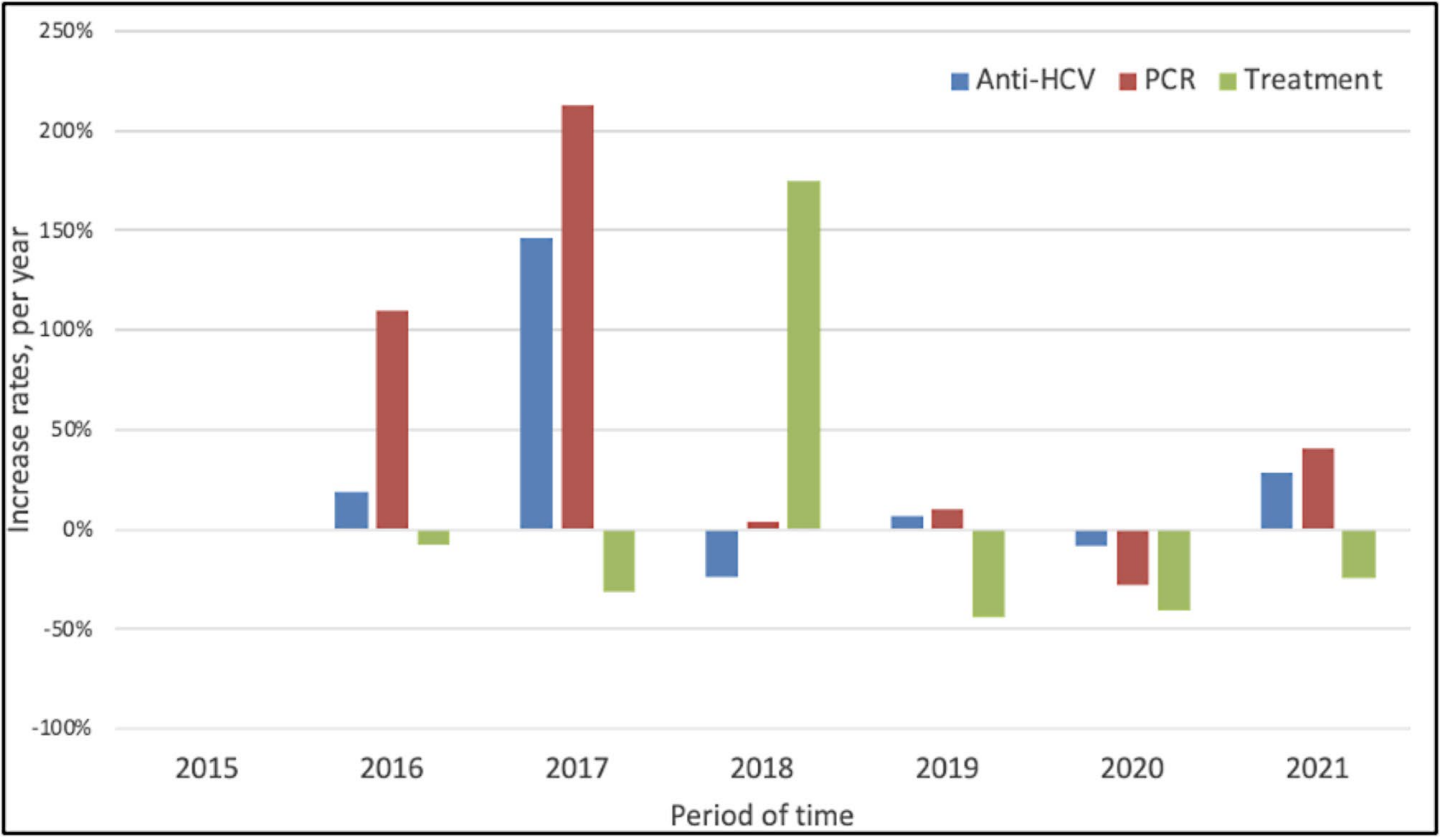
Annual percentage variation rate for HCV-Ab screening test (Anti-HCV), HCV diagnostic test (HCV RNA), and DAA treatment, Tuscany, 2015–2021

The graph in Fig. 5 shows the annual percentage variation rate in the uptake of HCV cascade of care services, namely: HCV screening test, HCV diagnostic test and DAA treatment. Concerning HCV screening and diagnostic tests, there was an increasing trend in 2016 and 2017, followed by a modest increase in the following years. Treatment prescriptions reached a peak annual percentage increase in 2018, then decreasing in subsequent years.

## Discussion

Here we describe the coverage and testing trends and access to treatment for HCV in Tuscany in the six years following the introduction of Direct-Acting Antivirals (DAA). To the best of our knowledge, this is the first attempt to monitor HCV services uptake following the introduction of DAA in Italy using a retrospective data-linkage approach. While building on previous studies conducted in the region, we highlight important weaknesses of the exclusive reliance on administrative healthcare records to monitor impact of largescale interventions.

Starting from 2015, there was a slight increase in the number of individuals who were initiated on treatment with DAA in Tuscany. A substantial increase in the number of HCV tests performed, close to 150% was observed in 2017 compared to 2016. This increase was probably the result of the decision to grant universal access to HCV treatment irrespectively of the grade of liver fibrosis [18]. Patients recall was widely implemented to promote treatment uptake among those who had been previously excluded. This is likely to have contributed to the number of tests carried out by the LHAs in Tuscany in 2017. Other initiatives at the local level might also have contributed to an increase in screening opportunities, however a lot of data is lacking. These activities subsequently led to a spike in treatment recorded in 2018. Since 2018, the local government started a comprehensive plan to eradicate chronic hepatitis C, involving all the LHAs accredited to prescribe DAA treatment. The target was to treat approximately 6,221 patients per year over a 3-year period [19].

However, according to our results, the target was not reached. A considerable contraction of HCV-related services was evidenced after 2018, which was further reduced in 2020 due to the pandemic. This slowdown may be due to the fact that, in the first few years after the introduction of DAA treatment, many patients already linked to care were easily accessed and treated, as observed by other authors [24]. Screening strategies implemented during the study period were likely insufficient to identify people with asymptomatic and unknown HCV infection.

The impact of the COVID-19 pandemic on the progress towards HCV elimination has been discussed in other studies with similar conclusions (25–26). Shakeri A. et al. [26] observed a decrease in DAA uptake in the early months of the pandemic in several countries, and other authors have highlighted the negative impact of the COVID-19 pandemic on screening and PCR test rates and treatment coverage (27–28). The pandemic clearly shifted priorities to the prevention and control of the spread of SARS-CoV-2 [29]. The potential impact of the COVID-19 pandemic on HCV eradication strategies was highlighted by Blach S. et al. [27] suggesting excesses in hepatocellular carcinoma cases and liver-related deaths in the years following the pandemic.

For the 33–53 age group, the population screened for HCV in the study period was predominantly female, despite HCV infection being also prevalent among males, in Tuscany [30]. Higher uptake of HCV screening among women is probably due to the recommended screening for women during the first trimester of pregnancy, in line with international guidelines [24]. However, uptake of HCV diagnostic tests was more common among males in line with the expected epidemiology of HCV infection. This also explains the higher percentage of treatment uptake among the male population in the study sample.

In terms of possible risk factors, people with problematic substance use constituted the largest share of tested and treated individuals, in line with the European epidemiology [31]. PWUD are widely recognized as a priority population for HCV screening and care [32]. According to our results, nearly 42% of PWUD had a screening test three times or more during the study period, reflecting the risk exposure, compared to 6.5% in the general population. However, these people may not be representative of all PWUD residing in Tuscany. We also included individuals with exemption code for problematic substance use, who are more likely to be linked to harm reduction (HR) services and engaged in HCV preventive and specialized care. However, not all PWUD may access harm reduction services or have received an exemption code, and regional-based estimates for this population group are lacking. This might be the case of individuals who had recently started injecting drugs [33] or among other groups with high risk behaviours [34]. In order to reach these individuals, point-of-care testing opportunities, community-based services, peer-led outreach, and integrated care are needed [35]. However, decentralized HCV models are still being used without any uniform strategies in various regions of Italy, including Tuscany [36]. Moreover, screening tests seem to be more favorably accepted by individuals attending harm reduction services and prisons, when providing a quicker outcome and being less invasive. Given the high HCV prevalence within these populations, it would be appropriate to directly use the test for HCV-RNA bypassing the screening test in order to have a faster diagnosis [37]. In these contexts, the HCV reflex test can have a significant impact, as demonstrated by other authors. Indeed, the use of POC assays is associated with reduced time from antibody test to treatment initiation and increased treatment uptake [38]. Furthermore, it can be argued that the use of reflex tests for HCV is an important tool to be exploited, particularly in settings where individuals’ linkage to care is challenging (e.g. harm reduction sevices, communities, prisons). Laboratory-based and clinic-based HCV reflex VL testing increased uptake and reduced time to HCV VL testing and may increase HCV linkage to care, as demonstrated by other authors [39].

In order to carry out a population-based screening campaign, in May 2021 [23] the Italian Ministry of Health targeted three population groups: all people who use drugs; all people living in prison; and all people born between 1969 and 1989. Our analysis shows that in Tuscany most residents belonging to the target birth cohorts were still to be tested at the beginning of 2022. Among PWUD and PLP, the testing coverage was higher, however a lack of accurate estimates of population size hindered precise assessments. The screening campaign started in 2023 in Tuscany and our study may provide a useful baseline to evaluate the impact of the regional screening programme in the future.

The use of administrative healthcare records to monitor the progress of the HCV elimination efforts against global targets is not entirely suitable. The use of linked administrative healthcare records ensures population-wide coverage and reduces the risk of participation and attrition biases, which are common in traditional cohort studies with people experiencing social disadvantages and exclusion such as PLP and PWUD. However, the lack of access to test results hinders the capacity of the system to estimate size of HCV-infected population and to track linkage to care for those with a diagnosis. As a consequence, assessing the cascade of HCV care [15] in Tuscany is challenging. This is in line with the situation at national level, whereby partial data are available, despite substantial progress in expanding HCV treatment coverage achieved in the country [40]. Yet, in a recent study using the same methodological approach, Yousafzai et al. [41] described the HCV care cascade after the introduction of DAAs in Australia by retrieving HCV test results through data linkage, showing the potential for such a monitoring tool when coupled with expanded access healthcare data. Furthermore, data on socioeconomic position and relevant clinical outcomes of individuals linked to HCV care pathway in Tuscany are not easily accessible using administrative healthcare records, prompting the use of proxy, e.g. dispensing of DAA treatment versus intake or cure. The utility of the available information for accurate monitoring of impact is substantially reduced as well as its potential to inform the design of future regional plans.

Yet, our findings show the value of administrative healthcare record linkages in describing the access to HCV care pathway for hard-to-reach individuals for whom participation in primary research can be challenging. At present, such a linkage is often resource intensive and time consuming, hindering efforts to monitor trends and evaluate interventions at the population level. Investments in routine and responsive linkages (also across multiple sectors), as shown elsewhere [42] is possible and might help improving the responsiveness of services and tailoring policy responses.

Our study as some additional limitations. We identified PWUD using exemption codes for data linkage purposes. However, the same code is associated with all substance use dependencies, and not specifically for intravenous drugs, thus probably resulting in an overestimation of PWUD in our study. On the other hand, linkage to care (e.g., HR services) in the Regional Healthcare System (SSR) is a requirement for receiving an exemption code, leading to a potential underestimation of actual PWUD residing in Tuscany during the study period.

In addition, our analysis started from 2015, when DAAs were introduced, and testing services provided before that year were not considered. Also, the use of administrative healthcare records does not provide information on tests performed by private providers (e.g., non-governmental organizations). This could have led to an underestimation of the total percentage of the Tuscan population who had undergone HCV screening at least once in their life.

## Conclusions

Our study assessed the progress towards HCV elimination in Tuscany following the introduction of the treatment with DAAs.

The national HCV elimination strategy (PNEV) and the regional plan issued in 2018 have been successful in streamlining HCV care services, resulting in an increased uptake of DAA treatment in Tuscany after 2015. Treatment uptake rose rapidly until 2018 and declined sharply after the advent of the COVID-19 pandemic. HCV testing coverage did not increase substantially during the study period, with the exception of 2017, likely leading to a stagnation in the number of new individuals treated after 2018.

The implementation of the one-time national HCV screening is key to ensuring the identification of unknown infections in asymptomatic individuals and curb the undiagnosed HCV fraction in Italy and in Tuscany. However, to effectively and accurately assess the progress and impact, better HCV-related data are needed. Population-based data-linkage studies like this one have great potential in monitoring interventions at the regional or national level, however they require comprehensive and timely access to administrative health records for epidemiological purposes.

### Electronic supplementary material

Below is the link to the electronic supplementary material.

Supplementary Material 1

## Data Availability

The authors confirm that the data supporting the findings of this study are available within the article [and/or] its supplementary materials.
