# Supplementary material for: Evaluating hepatitis C cascade of care surveillance system in Tuscany, Italy, through a population retrospective data-linkage study, 2015–2021
Source: BMC Infect Dis. 2024 Mar 29;24:362. doi: 10.1186/s12879-024-09241-z (PMC10979555; doi:10.1186/s12879-024-09241-z)
Supplement: Supplementary file 1 — Supplementary Material 1 [file 12879_2024_9241_MOESM1_ESM.docx]

**SUPPLEMENTARY MATERIAL**

S table - Summary of dataset characteristics

| Name | Identifier | Date of birth | Sex | PCR | Year PCR | EC PCR | LHA PCR | Treatment | Year treatment | LHA Treatment | Screening | Year screening | EC screening | LHA screening |
| --- | --- | --- | --- | --- | --- | --- | --- | --- | --- | --- | --- | --- | --- | --- |
| Type | Unique ID | MM/DD/YYYY | M/F | Y/N | Year | code list | code list | Y/N | year | code list | Y/N | year | code list | code list |
| Description | Unique anonymous ID | - | - | Polymerase chain reaction (PCR) test for HCV-RNA research | Year of PCR test | List of codes referring to specific exemptions correlated with PCR | List of codes referring to Local Health Authorities which registered the test | Treatment initiation | Year of treatment initiation | List of codes referring to Local Health Authorities which dispensed the treatment | Anti-HCV Ab research test (ELISA) | Year of anti-HCV test | List of codes reffering to specific exemptions correlated with anti-HCV test | List of codes referring to Local Health Authorities which registered the test |
